# Asthma and Rhinitis Induced by Selective Immediate Reactions to Paracetamol and Non-steroidal Anti-inflammatory Drugs in Aspirin Tolerant Subjects

**DOI:** 10.3389/fphar.2016.00215

**Published:** 2016-07-20

**Authors:** Diana Pérez-Alzate, Natalia Blanca-López, Inmaculada Doña, José A. Agúndez, Elena García-Martín, José A. Cornejo-García, James R. Perkins, Miguel Blanca, Gabriela Canto

**Affiliations:** ^1^Allergy Service, Infanta Leonor University HospitalMadrid, Spain; ^2^Allergy Unit, IBIMA-Regional University Hospital of Malaga-UMAMálaga, Spain; ^3^Department of Pharmacology, University of ExtremaduraCaceres, Spain; ^4^Research Laboratory, IBIMA-Regional University Hospital of Malaga-UMAMálaga, Spain

**Keywords:** hypersensitivity, paracetamol, non-steroidal anti-inflammatory drugs, immediate allergy, asthma, rhinitis

## Abstract

In subjects with non-steroidal anti-inflammatory drugs (NSAIDs)- exacerbated respiratory disease (NERD) symptoms are triggered by acetyl salicylic acid (ASA) and other strong COX-1 inhibitors, and in some cases by weak COX-1 or by selective COX-2 inhibitors. The mechanism involved is related to prostaglandin pathway inhibition and leukotriene release. Subjects who react to a single NSAID and tolerate others are considered selective responders, and often present urticaria and/or angioedema and anaphylaxis (SNIUAA). An immunological mechanism is implicated in these reactions. However, anecdotal evidence suggests that selective responders who present respiratory airway symptoms may also exist. Our objective was to determine if subjects might develop selective responses to NSAIDs/paracetamol that manifest as upper/lower airways respiratory symptoms. For this purpose, we studied patients reporting asthma and/or rhinitis induced by paracetamol or a single NSAID that tolerated ASA. An allergological evaluation plus controlled challenge with ASA was carried out. If ASA tolerance was found, we proceeded with an oral challenge with the culprit drug. The appearance of symptoms was monitored by a clinical questionnaire and by measuring FEV1 and/or nasal airways volume changes pre and post challenge. From a total of 21 initial cases, we confirmed the appearance of nasal and/or bronchial manifestations in ten, characterized by a significant decrease in FEV1% and/or a decrease in nasal volume cavity after drug administration. All cases tolerated ASA. This shows that ASA tolerant subjects with asthma and/or rhinitis induced by paracetamol or a single NSAID without skin/systemic manifestations exist. Whether these patients represent a new clinical phenotype to be included within the current classification of hypersensitivity reactions to NSAIDs requires further investigation.

## Introduction

Non-steroidal anti-inflammatory drugs (NSAIDs) are the most frequent elicitors of drug hypersensitivity reactions (DHRs) (Doña et al., 2012; Jares et al., 2015), being able to provoke reactions with immunological and non-immunological mechanisms (Cornejo-Garcia et al., 2009). NSAID-DHRs are now considered more frequent than betalactam (BL) DHRs, which largely induce immunologically mediated reactions (Antúnez et al., 2006b).

Since the asthma triad was first reported (Widal et al., 1993; Samter and Beers, 1968), a considerable amount of work has been dedicated to the study of ASA-induced airways disease (Pleskow et al., 1983; Szczeklik et al., 2001; Stevenson, 2004). ASA and other NSAIDs can induce various clinical entities via two major mechanisms (Kowalski et al., 2011; Asero et al., 2013; Stevenson and Kowalski, 2013). The first mechanism is pharmacological, where individuals react to chemically unrelated NSAIDs (cross intolerance), whereas the second mechanism is immune-mediated, thought to be related to specific IgE antibodies or sensitized T cells (selective reactions, SRs) (Kowalski et al., 2013). Entities in the former category are generally more frequent (Canto et al., 2009; Doña et al., 2011, 2012) and pharmacogenetic studies have been conducted for patients with respiratory airways (Ayuso et al., 2015) and cutaneous involvement (Cornejo-García et al., 2012, 2013). These studies are opening new avenues toward an understanding of the mechanisms involved (Perkins et al., 2015).

NSAIDs-exacerbated respiratory disease (NERD) belongs to the cross intolerance category and consists of chronic rhinosinusitis, nasal polyposis and/or bronchial asthma. These entities may appear in isolation but usually present together (Stevenson, 2004; Kowalski et al., 2013). They are aggravated by ASA and other strong COX-1 inhibitors, although intolerance of weak COX-1 and selective COX-2 inhibitors can also occur (Pleskow et al., 1983; Szczeklik et al., 2001; Stevenson and Kowalski, 2013). Although these patients may initially develop symptoms with different NSAIDs, a hallmark of NERD is the elicitation of a reaction after ASA intake (Asero et al., 2013; Kowalski et al., 2013).

Paracetamol is also responsible for respiratory reactions and can be a risk factor for asthma (Blanca-López et al., 2015) development, although the underlying mechanisms are currently unknown (Lesko, 2002).

Few studies have examined individuals who react to a single NSAID or paracetamol but tolerate ASA (Corominas et al., 2012). Nevertheless, the existence of this type of response has been shown (Cavkayter et al., 2015). One of the problems when studying this entity is that respiratory airways involvement may occur during an anaphylactic reaction to a single NSAID (Doña et al., 2011; Ayuso et al., 2013).

In this study, we present a series of cases who reported isolated upper and/or lower respiratory symptoms after taking a COX-1 or selective COX-2 inhibitor, but tolerated other NSAIDs including ASA. Diagnosis was confirmed by controlled challenge with the culprit drug and proving ASA tolerance.

## Materials and Methods

### Patient Selection

Patients reporting a respiratory reaction but no other symptoms after NSAIDs or paracetamol intake were evaluated in two centers integrated into the Spanish National Network for the Study of Drug and Allergen Hypersensitivity (Infanta Leonor University Hospital in Madrid, and Regional University Hospital of Malaga). A prerequisite for inclusion was that the patient reported ASA tolerance – this was then confirmed by ASA challenge in this study. In addition, challenge with the culprit drug was performed to confirm the diagnosis of a selective respiratory reaction (Doña et al., 2011, 2012). We did not include subjects with chronic spontaneous urticaria, nasal polyposis, respiratory infections, autoimmune diseases, pregnancy or who were undergoing breast-feeding.

This study was performed according to the principles of the Declaration of Helsinki and approved by the local ethics committee from the Infanta Leonor University Hospital and the Regional University Hospital of Malaga. All patients were informed orally about the study and signed the corresponding informed consent.

### Clinical Evaluation

Patients were subjected to a clinical questionnaire, skin prick testing (SPT) and had total serum IgE levels measured. Nasal and bronchial symptoms were assessed as described (Doña et al., 2011; Campo et al., 2013). Symptoms attributable to foods, hymenoptera sting and potential drug allergies were also recorded. A panel of 30 common inhalant and food allergens prevalent in the area of study was used for skin testing. A wheal diameter larger than 3 mm was considered positive. A negative saline control was also used.

Nasal acoustic rhinomanometry was performed and basal FEV1 estimated (Campo et al., 2013). Nasal exploration including a CT scan was also performed to exclude patients with polyposis, chronic rhinosinusitis or any other underlying nasal disease.

### Nasal Challenge with Lysine-Aspirin (lys-ASA)

Nasal challenge with lys-ASA was performed as described (Lee et al., 2004; Nizankowska-Mogilnicka et al., 2007). The appearance of nasal symptoms plus a decrease of 30% in the total volume of both nasal cavities measured by acoustic rhynometry (AR) was considered a positive result. The response of the lower airways after nasal challenge was also monitored by FEV1. A decrease greater than 12% compared to basal values was considered positive (Campo et al., 2013).

### Oral Provocation with ASA

This was performed using a single blind procedure as described (Doña et al., 2011), with the following modifications: on the first day, we administered placebo and if no reaction occurred, increasing oral concentrations of ASA were given at intervals of 90 min (doses of 25, 100, and 250 mg; cumulative dose of 375 mg). If no symptoms appeared, we repeated the challenge 1 week later but with a starting dose of 500 mg of ASA; if this was tolerated another 500 mg dose was given 90 min later (cumulative dose of 1000 mg).

### Oral Provocation with the Culprit Drug

When using the suspected culprit drug, we started with lower concentrations, following previous recommendations (Doña et al., 2011). For ibuprofen, increasing doses of 5, 20, 75, 150, and 350 mg were given at 45-minute intervals. If symptoms appeared at any time the procedure was stopped immediately. Clinical symptoms were assessed and changes in nasal flow were monitored by acoustic rhynomanometry; lower airways involvement was monitored by FEV1. For paracetamol the same approach was used but with doses of 5, 20, 50, 75, 150, and 200 mg. If symptoms did not appear, another 500 mg of paracetamol were given (cumulative dose of 1000 mg). For rofecoxib, we used doses of 5, 15, 40, and 60 mg (cumulative dose of 120 mg), whereas for dexketoprofen, we used doses of 5, 20, and 25 mg (cumulative dose of 50 mg).

## Results

A total of 21 patients reporting symptoms of rhinitis and/or asthma after the intake of paracetamol or a single NSAID, but also reporting ASA tolerance were prospectively recruited from January 2010 to December 2014. In all patients ASA tolerance was also assessed by challenge as described in the Methods section.

After completing the study, nasal and/or lower airways symptoms attributable to the intake of the culprit drug could be confirmed for 10 patients (**Table 1**). There was no clear difference in gender (four were female; six male). Their ages ranged from 22 to 69 years old, with a mean of 51.5. In terms of atopy, six were atopic and four non-atopic. The mean number of episodes was 4.3, although this value ranged from 2 to 10 amongst patients.

**Table 1:** Clinical characteristics and drugs involved.

Patient No.	Sex	Age (years)	Atopy	Positive allergen SPT	Serum specific IgE (kU/l)	Culprit drug	No. of episodes	Time interval (min)	Reaction
1	F	47	+	*Phleum pratense* *Lollium perenne*	2.15 1.65	Dexketoprofen	3	20–30	Asthma
2	F	22	+	*Lollium perenne*	1.55	Etoricoxib	3	30–60	Rhinitis
3	F	69	+	*Alternaria alternata* *Aspergillus fumigatus*	1.31 11.7	Ibuprofen	6	30–60	Rhinitis
4	M	38	+	*Dermatophagoides* *pteronyssinus* *Olea europaea*	1.25 1.95	Ibuprofen	7	60–120	Rhinitis + Asthma
5	M	65	-	NA	NA	Ibuprofen	3	60–120	Rhinitis + Asthma
6	F	47	+	*Dermatophagoides* *pteronyssinus* *Alternaria alternata* *Aspergillus fumigatus*	2.34 1.25 1.45	Ibuprofen	4	60–120	Rhinitis + Asthma
7	M	66	-	NA	NA	Paracetamol	2	20–30	Asthma
8	M	50	+	*Olea europaea* *Lollium perenne* Cat dander	1.23 3.23 1.60	Paracetamol	10	20–30	Asthma
9	M	35	-	NA	NA	Paracetamol	3	30–60	Asthma
10	M	56	-	NA	NA	Paracetamol	2	30–60	Rhinitis

*NA, not applicable.*

Ibuprofen was involved in four cases, paracetamol in 4, desketoprofen in 1 and etoricoxib in 1 (**Table 1**). Most patients reported three or more previous episodes (except patients 7 and 10, who reported only 2 previous episodes). Rhinitis, with or without asthma, appeared in six patients whereas isolated asthma occurred in four. Concerning atopic status, six patients showed a positive SPT to more than one common inhalant allergen (**Table 1**). No positive SPT to food allergens was observed. Clinical entities and the time interval elapsed between drug intake and appearance of symptoms according to patient history are provided in **Table 1**, whereas **Table 2** shows the challenge results.

**Table 2:** Results of challenge: time intervals between drug administration and the appearance of clinical symptoms, last and cumulative doses, and clinical symptoms induced.

Patient No.	Culprit drug	No. of episodes	Time interval (min)	Reaction
1	Dexketoprofen	3	20–30	Asthma
2	Etoricoxib	3	30–60	Rhinitis
3	Ibuprofen	6	30–60	Rhinitis
4	Ibuprofen	7	60–120	Rhinitis + Asthma
5	Ibuprofen	3	60–120	Rhinitis + Asthma
6	Ibuprofen	4	60–120	Rhinitis + Asthma
7	Paracetamol	2	20–30	Asthma
8	Paracetamol	10	20–30	Asthma
9	Paracetamol	3	30–60	Asthma
10	Paracetamol	2	30–60	Rhinitis

*AR, acoustic rhynometry; FEV1, forced expiratory volume in 1 s; DPT, drug provocation test.*

Although statistical comparisons were not performed due to the limited number of patients, we observed a tendency for ibuprofen to induce a reaction at higher doses and after longer time intervals compared to paracetamol (**Table 2**).

Concerning the cumulative dose required to elicit the reaction, this was variable for each drug (**Table 2**). For three patients the response to ibuprofen appeared at a dose of less than half the therapeutic dose; this also occurred in three patients who reacted to paracetamol.

## Discussion

In this study, we have presented for the first time a series of patients with SRs to paracetamol or a single NSAID with exclusive respiratory airway involvement. The approach for identifying these patients was based on clinical history, negative challenge with ASA and positive challenge with the culprit drug. Of the 21 cases initially considered, ten could be confirmed as tolerating asthma but reacting to the culprit drug. On average, these patients were older than the eleven other cases and reported more previous episodes.

Selective reactions to NSAIDs have been reported by several groups and for all available NSAIDs and selective COX-2 inhibitors (reviewed in Canto et al., 2009; Cornejo-Garcia et al., 2009; Blanca-Lopez et al., 2014; Torres et al., 2014). Pyrazolones, although not considered NSAIDs, are common triggers (Kowalski et al., 2013; Demir et al., 2015); other important drugs include diclofenac (Gala et al., 1998; Del Pozo et al., 2000; Harrer et al., 2010; Picaud et al., 2014), ibuprofen (Koransky et al., 2016), as well as weak COX-1 (Vidal et al., 1997; Astarita et al., 2011), and COX-2 inhibitors (Fontaine et al., 2005; Chamberlin and Silverman, 2009). In fact, in some countries SRs are responsible for up to 50% of all NSAID-DHRs (Demir et al., 2015). However, in these reported cases the symptoms induced were anaphylaxis and/or urticaria, and in those reactions with respiratory airway involvement, other organs were also implicated (Doña et al., 2011). All patients included in this study developed respiratory symptoms only, i.e., nasal symptoms and/or AR/FEV1 decreases, without other organ involvement.

For pyrazolones, positive skin and/or basophil activation test results have been found with metamizole (Gomez et al., 2009), providing indirect evidence of a potential IgE-mediated reaction. However, the only pyrazolone for which specific IgE-mediated reactions have been demonstrated is propyphenazone (Himly et al., 2003). Positive skin tests results for other NSAIDs are almost anecdotal with very few cases reported (Canto et al., 2009). Consequently, we did not perform skin testing with the culprit drug here.

Considering cross intolerance reactions, patients may present both respiratory and cutaneous involvement (blended reactions) (Stevenson and Kowalski, 2013), which have been reported as being more frequent than cases with exclusively respiratory symptoms (Doña et al., 2011). Questions remain as to whether blended reactions constitute a well-defined entity or a more heterogeneous group. Our feeling is that this entity includes different phenotypes (Ayuso et al., 2013).

Concerning respiratory SRs, only one case has been reported in the literature so far (Corominas et al., 2012). Repeated episodes of bronchospasm induced by paracetamol occurred in a 19-year-old atopic woman, who tolerated 500 mg of ASA. After the intake of 500 mg of paracetamol she presented a severe episode of bronchospasm with a decrease in FEV1 that required treatment with salbutamol (Corominas et al., 2012). Although the existence of respiratory SRs has been suggested previously (Cavkayter et al., 2015), this pattern of reaction has not been clearly identified. The identification of these reactions may be complicated due to respiratory symptoms being accompanied with mild symptoms in other organs: these reactions may be hard to distinguish from anaphylactic reactions. In fact, in IgE-mediated DHRs an initial mild reaction can be followed by a more severe one after subsequent exposure (Antúnez et al., 2006a). This phenomenon is thought to be due to greater levels of IgE antibodies and increased affinity. For the patients reported here, no evidence for increasing reaction severity was found.

Summarizing, in this study, we have presented a group of patients with an unusual pattern of response, which does not fit properly in the current classification system of DHRs to NSAIDs (Ayuso et al., 2013; Kowalski et al., 2013; Agúndez et al., 2015). Further attempts are underway to identify more patients with these characteristics and potentially establish the mechanisms involved.

## Author Contributions

NB-L, JC-G, MB, and GC conceived the study, revised protocols and data, and wrote the manuscript. DP-A, MB, NB-L, and ID selected all patients, performed the allergological workup and analyzed data. JA, EG-M, and JP analyzed data and revised the manuscript. JP reviewed the final English version. All the authors read and approved the final manuscript.

## Conflict of Interest Statement

The authors declare that the research was conducted in the absence of any commercial or financial relationships that could be construed as a potential conflict of interest.
